# Investigation of Three-Dimensional Flow Around a Model Samara Wing Depending on the Angle of Attack

**DOI:** 10.3390/biomimetics11050299

**Published:** 2026-04-25

**Authors:** Neslihan Aydın, Ebubekir Beyazoglu, Irfan Karagoz

**Affiliations:** 1 Mechanical Engineering Department, Engineering Faculty, Bursa Uludag University, 16059 Bursa, Turkey; neslihanaydin@uludag.edu.tr (N.A.); ebeyazoglu@uludag.edu.tr (E.B.); 2 Ulutek Technopark, 16285 Nilüfer-Bursa, Turkey

**Keywords:** bio-inspired wing, wing aerodynamics, computational fluid dynamics (CFD), flow separation and recirculation, lift and drag, wind turbines

## Abstract

One of the engineering applications inspired by nature is bio-inspired wings. The aerodynamic properties and autorotation characteristics of samara wing models have been studied extensively using both experimental and numerical methods. However, the three-dimensional flow behavior and angle of attack interaction around a natural samara wing are not yet fully understood. This study investigates the flow behavior around a samara wing model, with the aim of underlying physics and qualitatively analyzing the flow field, as well as the aerodynamic forces and stresses. Since the samara wing and the flow around it are three-dimensional, the difficulty of experimental investigation was taken into account, and the numerical analysis was performed using Computational Fluid Dynamics techniques. The results obtained from the numerical solution of the governing equations for three-dimensional turbulent flow were verified with experimental data. The calculations were performed by varying the angle of attack of the model wing between 0 and 50 degrees at 10-degree intervals. Depending on the angle of attack, the velocity field around the wing, surface pressure, and stress distributions, vortex structures formed on the wing and streamlines were analyzed, and the results were presented. This study and its results on this model may lead to the development and optimization of the model and its use in turbines or air vehicles.

## 1. Introduction

Biomimicry, which has been known for a long time, but its scientific basis has been developed in recent years, is an innovative approach that seeks sustainable solutions to human problems by taking inspiration from natural structures and strategies. Consequently, biomimicry has become a new branch of science, transferring ideas from biology to architecture, technology and many other disciplines. In recent years, many industrial designs inspired by nature have been realized and made available to people in the fields of flow control, material production, construction and shipbuilding, medical and engineering, aerospace and aircraft design, wind and solar energy use, and architectural designs [1,2,3,4,5,6]. Bio-inspired designs can also help to reduce the negative impact on the environment.

An important part of the work on bio-inspiration is wing development studies. These studies can be examined under several groups. One of them is to investigate the effects of leading-edge protuberances inspired by the fin of a humpback whale. The numerical results reported by Li et al. show that the lift coefficient may be increased by 5–15%, while the drag coefficient is significantly reduced with the spacing between the protuberances on the leading edge of the wing [7]. On the other hand, the numerical results reported by Li et al. [8] show that increasing the amplitude of the leading-edge protuberances can reduce the pressure amplitude, significantly reduce the cavitation volume, and improve the cavitation suppression. Cai et al. [9] tested NACA airfoils with a single leading-edge protuberance of indifferent amplitude in a wind tunnel to investigate the aerodynamic performance and flow separation characteristics. The results showed that all the modified airfoils exhibited a special two-step stall performance. Recently, a numerical study aiming to provide a more comprehensive picture of the flow dynamics of the leading-edge protuberances confirmed the existence of the compartmentalization effect caused by leading-edge protuberances [10].

Airfoils with leading-edge serrations inspired by owls are also studied for aeroacoustic properties. NACA0012 airfoils with leading-edge serrations and surface ridges were reconstructed and studied to reveal their noise reduction mechanisms. The results showed that airfoil with leading-edge serrations can reduce the sound pressure level by 14.3 dB [11]. Studies of bio-inspired trailing-edge structures have focused on their effects on noise and wake dynamics. Hasheminasab et al. [12] investigated the effect of trailing-edge serrations on airfoil performance and wake formation. Experimental results showed that serrations significantly increase the velocity fluctuations as well as the turbulent kinetic energy in the wake region of the cylinder and concentrate this energy within the three identified mode pairs. The flow around a NACA profile was studied using direct numerical simulation at Reynolds number 50,000 and angle of attack of 5° in cases of straight, blunt and serrated trailing edges. The flow pattern in the wake region, the shear layer and the vortex shedding characteristics were investigated comparatively [13]. Recently, Ma et al. [14] proposed biomimetic trailing-edge serration to reduce flow noise from submarines.

The noise reduction mechanism of airfoils with asymmetric and conventional trailing-edge serrations inspired by the owl wing was investigated at a low Mach number. The best results in terms of aerodynamic performance and noise reduction are achieved with oblique and asymmetric trailing-edge serrations [15]. Sinusoidal serrations applied to the leading edges of a flat plate and a NACA airfoil were investigated for noise reduction. Noise reduction was found to be more sensitive to the amplitude compared to the wavelength of the serration. Noise reduction was found to be significantly higher for the flat plate, especially at higher frequencies [16]. Similarly, Rong and Liu [17] studied the specific morphological effects of leading-edge serrations and trailing-edge fringes on aeroacoustic performance, separately. They presented a numerical study of the aeroacoustic characteristics of owl-inspired trailing-edge fringes and their interaction with leading-edge serrations for sound radiation. Trailing-edge fringes were found to suppress flow separation and vortex shedding in the vicinity of the trailing edge, consequently reducing local velocity fluctuations and sound pressure levels. The results also showed that leading-edge serrations and trailing-edge fringes complement each other as an effective noise-reducing design. Li et al. [18] numerically investigated the hydrodynamic performance and flow noise characteristics of a hydrofoil with a wavy leading edge. The results show that a wavy leading edge can reduce the pressure fluctuation on the hydrofoil surface, which directly contributes to noise reduction.

The effects of bio-inspired blade surface structures have also been investigated. A bionic blade inspired by the airfoil of an owl and the herringbone groove structure of owl feathers was designed and applied to horizontal-axis wind turbines [18]. The results showed that the herringbone groove structure improved the flow attachment by generating vortices, which reduced the pressure on the leeward surface of the bionic blade and increased the output power. Dragonfly inspired corrugated airfoils were generated and numerically tested to determine the effect of corrugations lying over the surface along the chord length on both the upper and lower surfaces [19]. The numerical results showed that corrugations on the upper surface have little effect, while those on the lower surface cause lift enhancement, and a fully corrugated pressure side gives the best performance at attack angles of 9.79° and 14.83° at low Re. Hui et al. [20] investigated the effects of both the morphing process and the non-continuous surface feature on the tip-vortex flow characteristics at Re = 87,000 for a wing model inspired by the primary feathers of bird wings.

In recent years, there have been many studies inspired by the movements and mechanisms of birds’ and insects’ wings, especially for the development of micro-air vehicles [21,22,23,24,25], which we will not discuss in detail here.

The maple samara seed is another natural structure whose aerodynamics is of interest due to the movement that it makes after breaking off from its branch. Maple seeds have excellent autorotational properties. They start spinning almost as soon as they leave the tree. It is observed that even seeds with badly shaped or badly damaged wings can spin easily in the air. As it falls, the heavier end of the wing causes it to spin in the air, slowing its descent and allowing the wind to carry the maple seed, sometimes for a mile or more. Researchers have been studying the autorotation property of maple seeds, focusing on their use as rotors.

In a numerical study, Holden et al. [26] investigated the performance of a wind turbine blade inspired by maple seeds. The power coefficient reached a maximum of 0.59. They also compared the lift coefficient values, which are important in blade design, for varied Reynolds values of Re = 2000–20,000. The maximum lift coefficient value reached 0.8 at Re = 10,000. Studies have also shown that the autorotation of maple seeds during falling provides extra lift force [27]. In addition, Chin and Lentink [28], in a review study, said that the eddy regions formed at the leading edge generate high lift for various species of birds, insects and plants.

In a study conducted in 2019, Zakaria et al. [29] experimentally investigated the free-fall velocities of maple seeds. The geometric properties and angular velocities of different maple seeds were determined in order to calculate their free-fall velocities. In contrast, Win et al. [30] simulated the samara maple seed and used its autorotation properties to test a new autorotating aircraft in a vertical wind tunnel, looking at how long it took to reach certain speeds. Yogeshwaran et al. [31] obtained the aerodynamic properties of the samara maple seed by modeling drop tests with the 3D printing method and testing the velocity profiles with the LEV (Large Eddy Vortex) method. On the other hand, Chu [32] designed a biomimetic wind turbine blade from the seeds of the Dryobalanops aromatic tree which is partly similar to maple samara seeds. He used OPENFOAM V.18.06 for CFD flow analysis. The power coefficient reached 0.46 in the analyses, which were conducted at different blade tip speed ratios.

Studies of samara wings have focused on their autorotation characteristics. However, there has been no detailed study of the causes and consequences of these aerodynamic properties. Moreover, the stationary wing condition has scarcely been studied in the literature. In recent years, the aerodynamic characteristics of a stationary samara wing model, as well as a symmetrical wing model inspired by samara wings, have been studied experimentally [33,34]. In the first of these studies, the performance of a wing model with a symmetrical profile, although similar to the maple samara wing, was investigated experimentally and numerically, while in the second study, the lift and drag coefficients of an enlarged model of the maple samara wing were investigated experimentally. As a continuation of these studies, but differing from them, this study examines the flow structure around a stationary wing inspired by maple samara, and, for the first time, the changes in this structure as a function of the angle of attack. Another innovative aspect of the study is the investigation of the possible effects of the seed part on the flow in maple samara wings, since there is no study in the literature on maple samara wings with such a wavy surface, including a thick seed part. Knowledge of the cause-and-effect relationships will lead to the design and optimization of the wing to achieve the desired characteristics.

## 2. Modeling

The aim of this study was to investigate the flow around a maple seed at rest, which has unique aerodynamic characteristics, at different angles of attack. The first step was to observe and model the general structures of the maple seed.

### 2.1. Geometric Model

**Figure 1:**
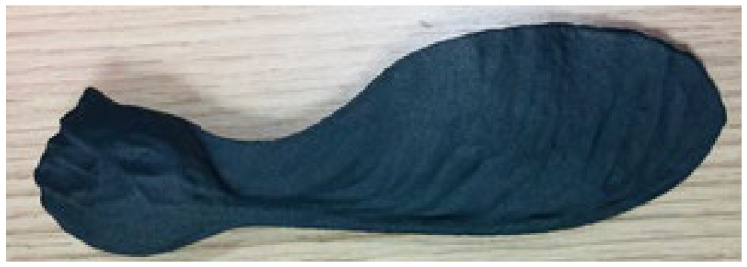
The prototype of a sample samara seed.

Although maple (samara) seeds have different structures and geometries, a typical seed found in nature was chosen for this study. The selected maple seed was scanned using an optical scanner and its 3D image was transferred to the computer environment and a cad file was created. A prototype of 16 cm in length was then produced using a 3D printer using polyamide material and multi-jet fusion technology. As can be seen in Figure 1, the model has been generated to include both the seed and leaf parts.

### 2.2. Mathematical Model

The mathematical model of the flow around the created prototype wing consists of the equations of conservation of mass and momentum of a three-dimensional turbulent flow. Since it was studied at low Mach numbers, the effect of compressibility was neglected in this study. The continuity equation and the Reynolds-averaged Navier–Stokes equations can be expressed as follows:

(1) $$\frac{\partial \rho }{\partial t}+\frac{\partial }{\partial x_{i}}(\rho u_{i})=0$$

(2) $$\frac{\partial }{\partial t}\left(\rho u_{i}\right)+\frac{\partial }{\partial x_{j}}\left(\rho u_{i}u_{j}\right)=-\frac{\partial \rho }{\partial x_{i}}+\frac{\partial }{\partial x_{j}}\;\;\left[µ\left(\frac{\partial u_{i}}{\partial x_{j}}+\frac{\partial u_{j}}{\partial x_{i}}-\frac{2}{3}\delta_{ij}\frac{\partial u_{i}}{\partial x_{j}}\right)\right]+\frac{\partial }{\partial x_{j}}\left(-\rho \bar{u_{ı}^{\prime }u_{ȷ\;}^{\prime }}\right)$$

Here $u_{i}$ is the velocity component and $\left(-\rho \bar{u_{ı}^{\prime }u_{ȷ\;}^{\prime }}\right)$ indicates the Reynolds turbulence stress. Due to the chaotic nature of turbulence, there are no analytical methods to calculate these values. Turbulence models are used to calculate the turbulence stresses in the momentum equation.

Different turbulence models were tested in this study. The standard k-ε turbulence model, the RNG k-ε turbulence model and the SST k-ω turbulence model with low Reynolds correction were tested, and the CFD results were compared with experimental data. The comparison between numerical results and experimental data is presented in Table 1. While all models capture the general trend, the SST k-w and Transitional SST models provided the closest agreement with experimental lift and drag coefficients. Among them, the SST k-ω turbulence model was used in subsequent analyses.

### 2.3. Numerical Model

**Figure 2:**
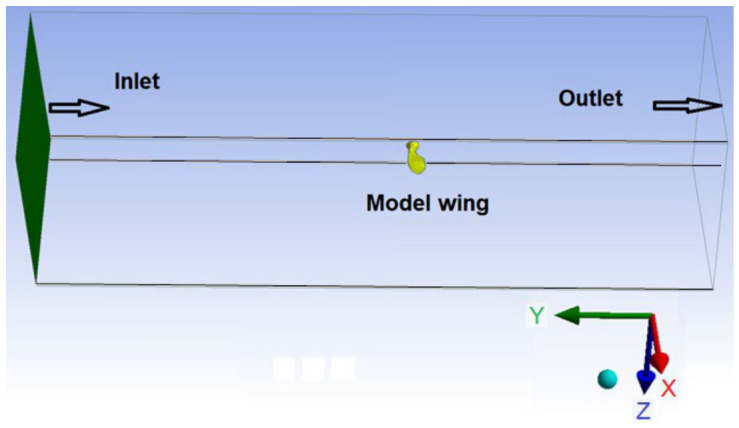
Computational domain.

The conservation equations of the flow are solved with the finite volume-based CFD software fluent 2025 R1 under the specified boundary conditions. Second-order discretization schemes were applied for momentum and turbulence equations, whereas a simplex algorithm was used for pressure–velocity coupling. The solution domain around the model wing was chosen to match the wind tunnel used in the experiments and has a cross-sectional area of 70 × 60 cm^2^ (Figure 2).

In the study of mesh independence, numerical tests were carried out on meshes with different numbers and structures ranging from 500 thousand to 4 million meshes. The results of the analysis showed that the results did not change much in the number of meshes above 2 million meshes. Based on the results obtained, approximately 2 million meshes would be considered sufficient for the accuracy of this study. These tests showed that the maximum relative error was approximately 1.85% for the optimum mesh configuration, which indicates a good agreement between the present results and those published in the literature. The quality of the generated mesh structures was evaluated. Skewness, which is particularly important for complex geometries, defines the deviation of the mesh from the actual plane of the model. Regarding mesh quality, while the average skewness is 0.6, we ensured that the maximum skewness in the critical boundary-layer regions remained below 0.25. To accurately resolve the boundary layer, 12 prism layers were used with a growth rate of 1.2, ensuring that the y+ values remained near one for the SST k-w model to function correctly. The convergence criterion is 10^−5^ for all the equations.

### 2.4. Boundary Conditions

The computational domain and boundaries considered are shown in Figure 2. The air enters the domain through the channel inlet with an atmospheric temperature of 20 °C and leaves the domain at the channel outlet. Uniform velocity distributions of 10 and 14 m/s were assumed in the inlet section. The outlet section is defined as the pressure outlet and the non-slip boundary condition is applied to the walls.

## 3. Results

In the computational domain created to study the flow around the maple wing whose characteristics are explained above, numerical solutions were obtained in the case of a free-flow velocity of V = 14 m/s, which corresponds to an Re number of about 34,000, based on the cord length. The purpose of choosing this speed is to be able to verify it with previously obtained experimental data and to simulate the flow around a maple wing in the natural environment. The solutions were repeated in 10-degree steps between 0 and 50 degree angles of attack. The verification results are presented first, followed by an examination of the structure of the flow around a stationary wing and its variation with angle of attack. Finally, the complex interaction between the three-dimensional separated flow beyond the maximum lift and the vortex shedding from the blunt trailing edge is revealed.

### 3.1. Validation

**Figure 3:**
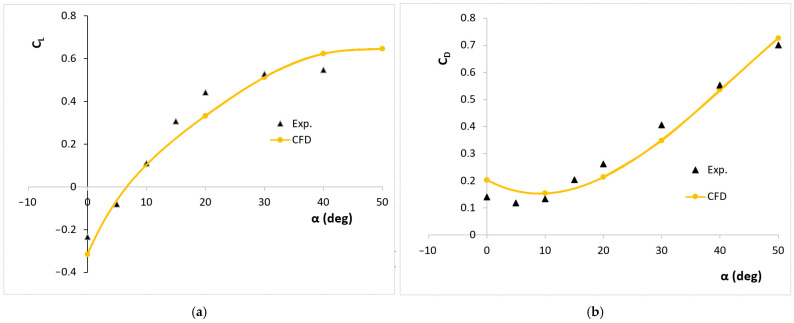
Computational and experimental [35] lift (**a**) and drag (**b**) coefficients vs. attack angle for the model wing.

Using the solution’s results, the lift and drag coefficients, which are defined in Equation (3), were calculated and their variations with angle of attack were determined. These numerical results are validated against previously published wind tunnel experiments [35]. The analysis shows that the spanwise extent of the computational domain is critical to the successful simulation of this flow, more so than the choice of modeling approach. In addition, a low drag regime observed at angles of attack prior to stall is identified and analyzed. Figure 3 presents a comparison of the computational and experimental lift and drag coefficients at a free-stream velocity of 10 m/s.

(3) $$C_{L}=\frac{F_{L}}{\frac{1}{2}\rho V^{2}A}\;,\;\;\;\;\;\;\;\;\;\;\;C_{D}=\frac{F_{D}}{\frac{1}{2}\rho V^{2}A}$$

It can be seen that there is generally good agreement with the experimental results. The angles of attack have been chosen to match the experimental data. Due to the undulating structure and the three-dimensionality of the wing shape, a negative lift coefficient was obtained at zero angle of attack, which is also in agreement with the experimental data. Again, zero lift coefficient was obtained at an angle of attack of about 8 degrees, which is also in agreement with the experimental data. The maximum lift coefficient was found to be 0.6, which is about 15% higher than the experimental data. The stall event is also seen around 50 degrees, with a little more delay compared to the experimental one. Looking at the drag coefficient, the minimum value in the experimental solution is around 8 degrees, while in the numerical solution it is seen at an angle of attack of 10 degrees. These results show that the zero angle of attack should be around 8 degrees. However, the undulating structure of the wing and its three-dimensionality make it difficult to find the zero angle of attack position. The angles of attack in this solution are assumed to be the same as the experimental angles. No comparison is made with the lift and drag coefficients of standard wing profiles. This is due to the significant influence of the wing root on the results.

Root Mean Square Error (RMSE) analysis was also performed to provide error quantification. The calculated RMSE value of 0.165 indicates the average deviation of the numerical (CFD) model from the experimental data. Especially for complex and asymmetrical geometries like a maple seed, this can be considered within acceptable limits. Despite the discrepancy in magnitude, the numerical model accurately captures the aerodynamic trends. The deviations and stall delay in numerical solution may indicate that the CFD model does not fully capture flow separation or turbulence effects at especially high attack angles. Possible causes for the discrepancies are wall effects, geometric simplifications, turbulence models and experimental uncertainties.

Unlike wind tunnel experiments, numerical simulations do not account for free-stream turbulence or surface roughness. Both of these factors are known to affect boundary-layer transition and flow separation. The use of the SST k-w turbulence model may delay separation due to its tendency to dampen turbulent fluctuations in certain flow regimes. This can lead to an overestimation of the lift force and a delay in stall onset. Although a sufficiently fine mesh structure was used, limitations in resolving gradients near the wall, especially higher attack angles, may contribute to the delay in stall prediction.

### 3.2. Velocity and Pressure Contours

**Figure 4:**
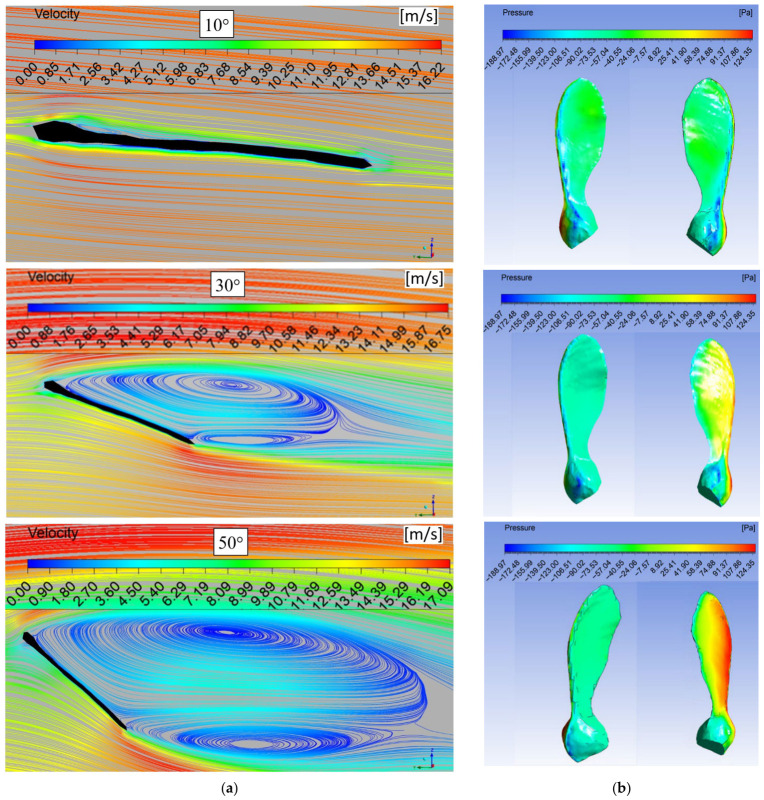
Velocity contours (**a**) at the middle section of the wing and pressure contours (**b**) on the bottom (**right**) and the top (**left**) surfaces of the wing for the free-stream velocity of 14 m/s.

The velocity contours in the wing’s mid-plane and the static pressure contours on the upper and lower surfaces of the wing are shown in Figure 4. At an angle of attack of 10 degrees, the streamlines are almost parallel and, as expected, the upper and lower surface boundary layers show a similar development. However, it is observed that a small leading-edge vortex (LEV) vortex forms in the pit area near the leading edge of the top surface. Due to this velocity field, the static pressure on the lower and upper surfaces of the wing does not show excessive changes. However, a low-pressure area forms along the leading edge of the wing as a result of the LEV. When the angle of attack reaches 30 degrees, the LEV expands to cover the wing surface, whilst a small secondary circulation region forms in the upper part of the trailing edge. Depending on this velocity distribution, as the pressure on the underside of the wing increases, the low-pressure region created by the LEV covers the upper surface of the wing, which contributes to an increase in lift. As the angle of attack increases, the LEV expands excessively on the wing, and the secondary vortex moves away from the wing. This also caused the pressure above the wing to rise slightly, while the bottom pressure continues to increase. This situation indicates that the stall is approaching. At high angles of attack, the lower surface pressure is almost uniform along the wing, while the lateral pressure distribution exhibits a distinct difference. The surface pressure distributions of the seed section are similar to those of the blunt body, but irregularities are seen due to surface curvature.

**Figure 5:**
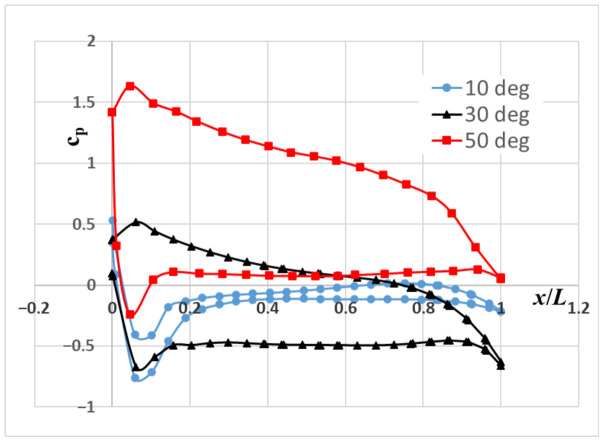
Variation in pressure coefficient along the cord length at the middle section of the wing.

The variation in the pressure coefficient in the wing’s mid-section plane is also examined. Figure 5 shows the variation in the pressure coefficient (Cp) along the chord length on the lower (pressure) and upper (suction) surfaces of the wing. As expected, the Cp distributions show a smooth pressure distribution across the surface, consistent with fully attached flow conditions at an angle of attack of 10 degrees. However, a low-pressure region forms in the area close to the leading edge of the wing due to the leading-edge vortex (LEV). While the pressure coefficient increases along the lower surface of the wing at a 30-degree angle of attack, except for the region near the trailing edge, it is clearly seen that as the angle of attack increases, a stronger suction peak is observed due to the LEV on the upper surface, accompanied by a gradual pressure recovery. As the angle of attack increases to 30°, the pressure difference between the upper and lower surfaces increases, confirming the increase in lift. Approaching stall conditions, the Cp distributions exhibit delayed pressure recovery on the suction side, indicating that flow separation occurs farther back, near the trailing edge. While the pressure coefficient increases significantly in the lower part of the wing at a 50-degree angle of attack, the pressure coefficient increases in the upper part of the wing compared to 30 degrees. The reason for this is the backflow formed near the wing surface due to the wide circulation area formed on the wing at high angles of attack.

### 3.3. Three-Dimensional Structure of the Flow

**Figure 6:**
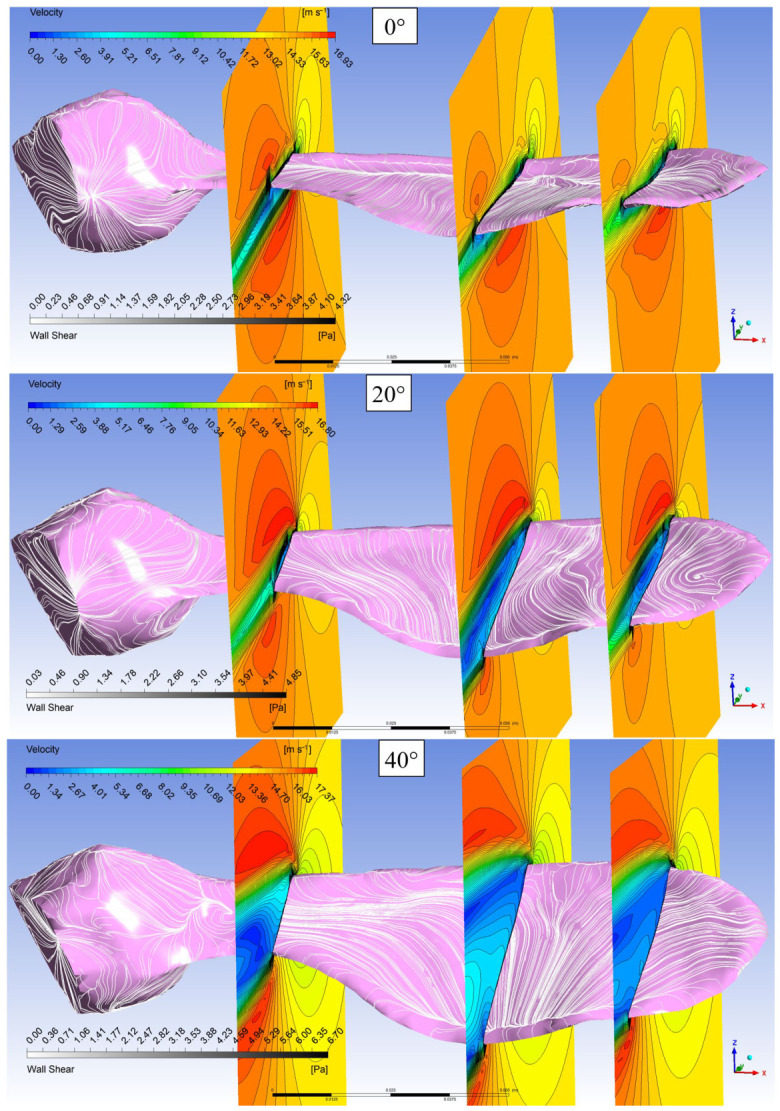
Streamlines on the wing surface and velocity contours in three different vertical planes for the free-stream velocity of 14 m/s.

Based on the results of the verified numerical solutions, the three-dimensional flow structure around the maple wing and its variation with the angle of attack are analyzed. For this purpose, in addition to the streamlines on the wing surface, velocity contours in three different vertical planes, namely the root, mid and tip, are shown in Figure 6. In the images taken at three different angles of attack, free flow is in the direction of the −*y* axis. The gray levels on the wing surface indicate the shear stress. Shear stresses will be discussed separately.

Figure 6 also provides a more detailed view of the separation and reattachment lines of the leading-edge vortex. As can be seen from the surface streamlines at zero angle of attack, the leading-edge vortex extends along the wing in a narrow region close to the leading edge; the separation and reattachment lines are clearly visible. In addition to the surface streamlines, the change in velocity contours also indicates the three-dimensional characteristics of the flow. It can be seen that the velocity contours in all three sections were almost parallel to the wing surface, especially in the boundary layer above the wing, but the velocity gradient in the boundary layer below the wing was higher. It can be said that this difference is due to the fact that the wings were not symmetrical and the wing surface was wavy. As the angle of attack increases, the LEV almost completely covers the wing surface, and the reattachment line extends to a region close to the trailing edge of the wing. This LEV structure maintains a high lift coefficient and delays stall as the angle of attack increases. On the other hand, the drag coefficient increases significantly with the angle of attack, leading to a rapid decrease in the Cl/Cd ratio.

From the velocity contours, especially in the mid and tip sections, it can be seen that a wide circulation region is formed in the upper part of the wing at a 20-degree angle of attack. The lowest velocities are observed at the centers of these circulation regions, while the maximum velocities occur just above the circulation regions. It can be seen that the surface flow lines continue approximately parallel to the cavities along the wing without merging in the cavity near the leading edge. A circulation core was formed in the region near the wing tip. This may indicate the presence of wing-tip vortices.

As the angle of attack continues to increase, it can be seen that the circulation areas on the wing are significantly widened and a smaller secondary circulation region is formed in the region close to the trailing edge. The surface streamlines again progress along the wing surface. At this angle of attack, it is noticed from the surface streamlines that the vortex at the wing tip is not formed, but a vortex is formed in the region close to the bottom. This is probably due to the flow directed by the seeds because of the low pressure formed on their wings.

### 3.4. Variation in Surface Shear Stresses

**Figure 7:**
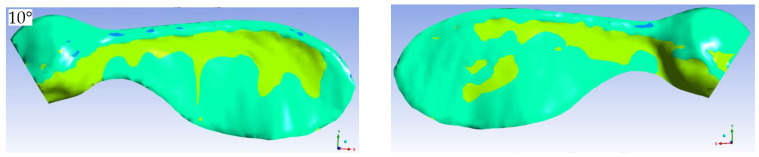

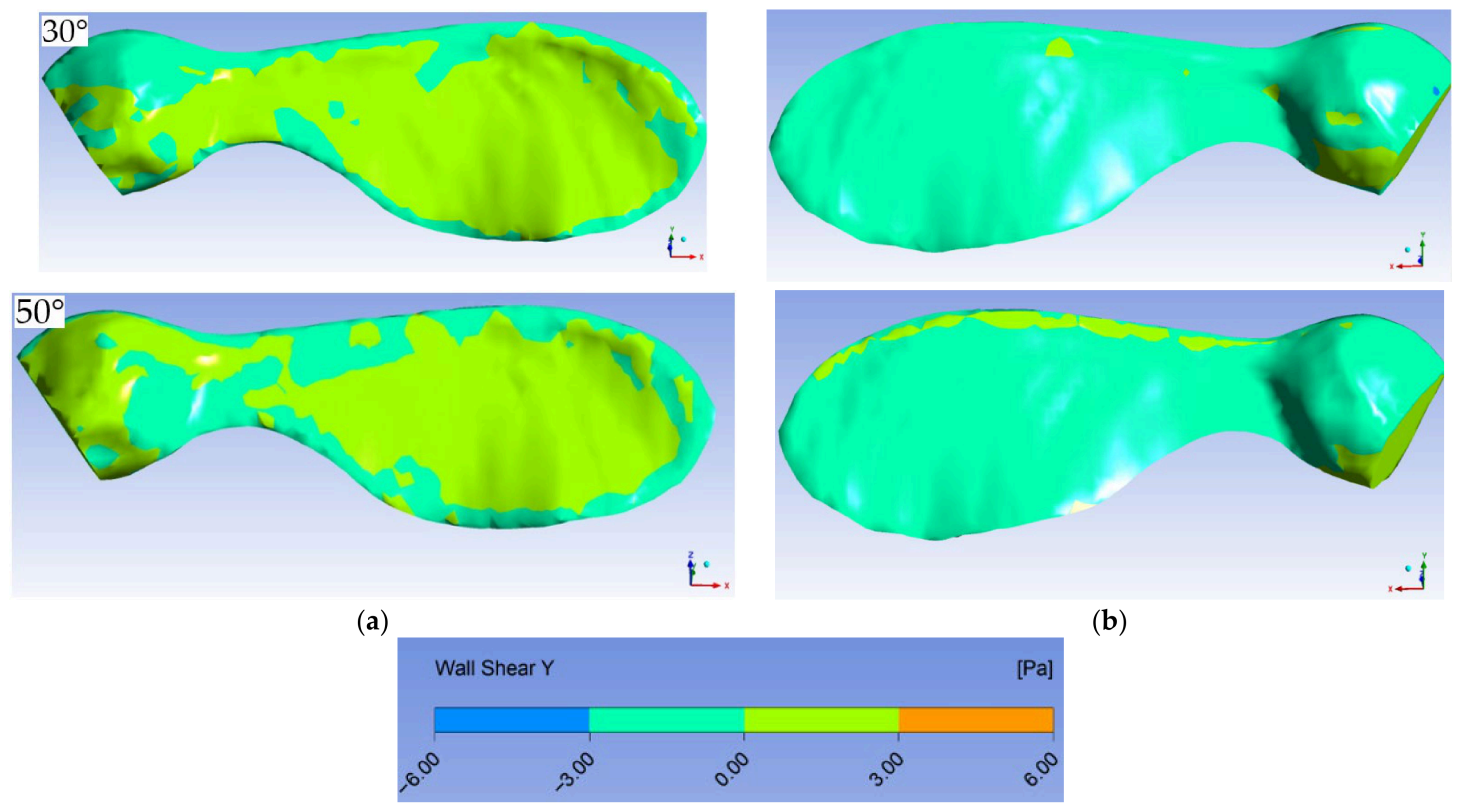
Contours of shear stresses in the y-direction on the upper (**a**) and lower (**b**) surfaces of the wing for the free-stream velocity of 14 m/s.

**Figure 8:**
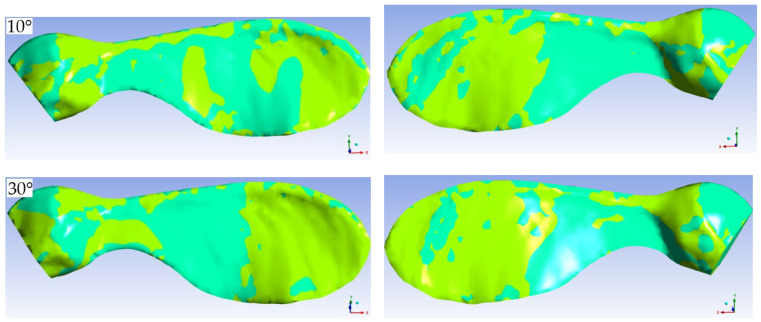

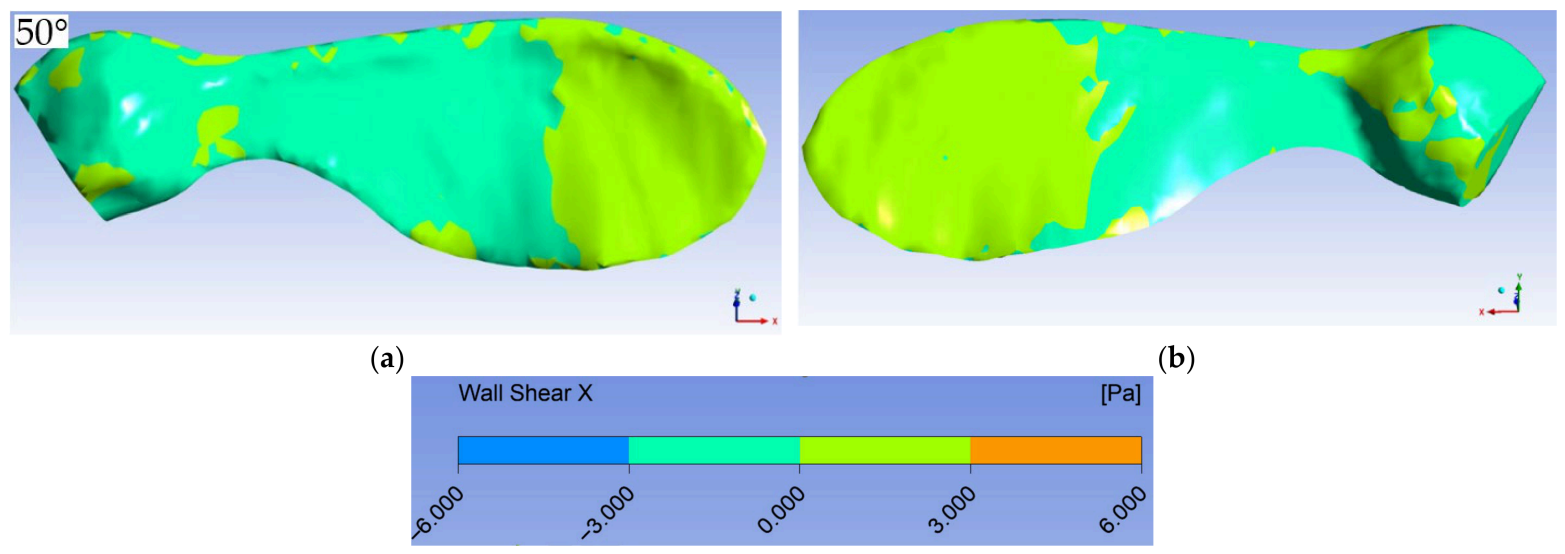
Contours of shear stresses in the *x*-direction on the upper (**a**) and lower (**b**) surfaces of the wing for the free-stream velocity of 14 m/s.

It is well known that shear stresses occur due to the viscosity of a fluid in a flow. For Newtonian fluids, these stresses are directly proportional to the velocity gradients. In this respect, the value of the stresses gives an idea of the magnitude of velocity gradients. In this study, contours showing the changes in surface shear stresses were drawn to examine the locations of the sign changes. The points or lines where the surface shear stresses change signs are important in terms of carrying information on the positions where the flow separates from the wall and reattaches to it. Figure 7 and Figure 8 show the shear stress contours in the y- and x-directions, respectively, at some angles of attack.

Figure 7, which shows the shear stress contours in the y-direction, reveals the separation and reattachment locations on both the lower and upper surfaces of the wing at an angle of attack of 10 degrees. On the upper surface, separation occurs close to the leading edge and reattachment occurs at the end of the circulation zone extending to the center of the wing. Separation and reattachment also occur on the underside of the wing, but in a narrower area. There is no separation on the upper and lower surfaces near the wing tip. Because the wing surface is not flat, the lines of separation and reattachment lines are not smooth.

At an angle of attack of 30 degrees, there is no separation and therefore no reunion on the lower surface of the wing, except for the seed part, and on the upper surface there is separation near the leading edge and reattachment near the trailing edge due to the expansion of the circulation zone. At a 50-degree angle of attack, there is separation and reattachment in a very narrow region near the leading edge on the lower surface, while separation is delayed on the upper surface, near the wing root.

Since the wing under consideration is three-dimensional and there is a three-dimensional flow, the circulation regions are also three dimensional. Figure 8 shows the variation in the x-component of the shear stresses. The regions where this component is zero and the sign of the stress changes can be considered a function of the angle of attack. As the angle of attack increases, the negative and positive stress regions on the lower and upper surfaces become more distinct and cover a larger area. These graphs also show that secondary flows in the plane perpendicular to the main flow create stagnation points on both the lower and upper surfaces of the airfoil. It can be said that this situation is due to the movement of the tip vortex towards the large low-pressure region that forms in the upper part of the wing as the angle of attack increases.

## 4. Conclusions

In this study, a natural samara wing model with unique aerodynamic characteristics was created and the behavior of the turbulent flow around it was numerically solved. The numerical solution was verified using experimental results from previous studies. The velocity and pressure distributions around the wing, the separation regions from the wall and the vortex structures were studied with the solutions obtained at different angles of attack between 0 and 50 degrees.

In addition to the uneven surface of the wing, the three-dimensional structure of the wing causes the flow around the wing to be complex and vortical. At low angles of attack, the weak LEV region at the leading edge grows with increasing angle of attack, especially in the center of the wing, and covers the upper surface of the wing, which eventually leads to a delayed stall. At high angles of attack, a second vortex region forms at the trailing edge and increases with the angle of attack. The pressure contours on the upper and lower surfaces of the wing become more uniform with increasing angle of attack, especially on the upper surface of the wing.

The separation and reattachment regions from the wall are investigated by means of the surface shear stresses. At low angles of attack, separation and reattachment from the wall occur on the lower and upper surfaces of the wing near the leading edge, while at high angles of attack, they occur only on the upper surface of the wing. Due to the three-dimensional structure of the flow, at high angles of attack, the x-direction shear stress due to wing-tip vortices creates distinct positive and negative regions. This can also contribute to autorotation.

In future studies, a similar wing without the seed part will be designed, analyzed, and optimized in order to develop a high-performance wing, particularly for systems with translational and rotational wings.

## Figures and Tables

**Table 1:** Comparison of the turbulence models.

Turbulence Model	C_L_	C_D_	Error in C_L_ (%)	Error in C_D_ (%)
Standard k-ε	0.417	0.236	29.5	28.3
RNG k-ε	0.430	0.228	33.5	23.9
SST k-w low Reynolds correction	0.323	0.206	0.3	11.9
Transitional SST k-w	0.379	0.210	17.7	14.1
Experimental	0.322	0.184		

## Data Availability

The data presented in this study is available on request from the corresponding author.
